# DeepIso: A Deep Learning Model for Peptide Feature Detection from LC-MS map

**DOI:** 10.1038/s41598-019-52954-4

**Published:** 2019-11-20

**Authors:** Fatema Tuz Zohora, M. Ziaur Rahman, Ngoc Hieu Tran, Lei Xin, Baozhen Shan, Ming Li

**Affiliations:** 1 David R. Cheriton School of Computer Science, University of Waterloo, Waterloo, ON N2L 3G1 Canada; 2 Bioinformatics Solutions Inc., Waterloo, ON N2L 6J2 Canada

**Keywords:** Machine learning, Proteome informatics

## Abstract

Liquid chromatography with tandem mass spectrometry (LC-MS/MS) based quantitative proteomics provides the relative different protein abundance in healthy and disease-afflicted patients, which offers the information for molecular interactions, signaling pathways, and biomarker identification to serve the drug discovery and clinical research. Typical analysis workflow begins with the peptide feature detection and intensity calculation from LC-MS map. We are the first to propose a deep learning based model, DeepIso, that combines recent advances in Convolutional Neural Network (CNN) and Recurrent Neural Network (RNN) to detect peptide features of different charge states, as well as, estimate their intensity. Existing tools are designed with limited engineered features and domain-specific parameters, which are hardly updated despite a huge amount of new coming proteomic data. On the other hand, DeepIso consisting of two separate deep learning based modules, learns multiple levels of representation of high dimensional data itself through many layers of neurons, and adaptable to newly acquired data. The peptide feature list reported by our model matches with 97.43% of high quality MS/MS identifications in a benchmark dataset, which is higher than the matching produced by several widely used tools. Our results demonstrate that novel deep learning tools are desirable to advance the state-of-the-art in protein identification and quantification.

## Introduction

The outstanding performance of deep learning on object recognition opens a new frontier in the domain of bioinformatics. As a continuation of that, our research on solving peptide feature detection problem using Convolutional Neural Network (CNN) and Recurrent Neural Network (RNN) is the first attempt as per our knowledge. The CNN is a type of feed-forward artificial neural network in which the connectivity pattern between its neurons is biologically inspired by the organization of the animal visual cortex^1,2^. CNN became popular after the revolutionary breakthrough in ImageNet^3^ object recognition competition, 2012. RNN uses the internal state (memory) to exhibit temporal dynamic behavior which brings groundbreaking results in many natural language processing tasks, for instance, Google Neural Machine Translation system^4^.

On the other hand, proteomics based on liquid chromatography with tandem mass spectrometry (LC-MS/MS) is a well-established research field for the discovery of disease biomarkers, drug target validation, mode of action (MOA) studies, and safety marker identification in drug research^5^. The latest advanced types of LC-MS technologies generate huge amounts of analytical data with high scan speed, and high resolution, which is almost impossible to interpret manually. Deep neural networks are found to be very effective and flexible to discover complex structures of the data through its many layers of neurons. Thus it has made its way into analyzing LC-MS data as well^6^. For instance, DeepNovo^7,8^ introduces deep learning to de novo peptide sequencing from tandem MS data. Bulik-Sullivan *et al*.^9^ creates a computational model of antigen presentation for neoantigen prediction using deep learning. DeepSig^10^ takes input protein sequence to detect signal peptides and predict cleavage-site. DeepRT^11^ provides improved peptide Retention Time prediction in liquid chromatography. The application of deep learning in this field is becoming a promising approach day by day.

Protein identification and quantification are fundamental tasks in proteomics and peptides are the building block of protein. Protein is first enzymatically digested into peptides, and then peptides are analyzed by LC-MS/MS instruments. Typical analysis workflow of LC-MS/MS data includes peptide feature detection and intensity calculation from an LC-MS map, peptide identification from MS/MS spectra, and protein profiling^12–14^. The first step, peptide feature detection and intensity calculation from an LC-MS map is our target problem. The LC-MS map of a protein sample is a 3D plot where the three dimensions are: mass-to-charge (*m*/*z*) or Da, retention time (RT), and intensity (I) of peptide ions in that sample. Peptide feature is a multi-isotope pattern formed by different molecular isotopes, e.g. carbon-12 and carbon-13, of the same peptide. Detecting multi-isotope patterns in LC-MS map is a challenging task due to the overlapping peptides, several charges of the same molecule, and intensity variation. Moreover, a single LC-MS map may have gigapixel size containing thousands to millions of peptide features. However, CNN is found to be effective in similar pattern recognition problems, for example, in detecting cancer metastasis on gigapixel pathology images by Liu *et al*.^15^. Moreover, a combination of RNN with CNN can deal with the pattern spanning over multiple time frames. For instance, video classification^16^ using FC-RNN model. Detecting peptide features in a highly sparse and noisy LC-MS map can relate to the problem of selecting frame of interests in a noisy and unsegmented sequence, which can be handled using temporal attention-gated model^17^. Therefore, to address our target problem, we propose a deep learning based model DeepIso to detect peptide features along with their charge states and estimate their intensities in a LC-MS map. It works in two steps. In the first step, IsoDetecting module spots the multi-isotope patterns and generates a list of detected isotopes. In the second step, IsoGrouping module goes around the spotted region of interests, and groups multiple isotopes into a peptide feature. Our initial work is described in a technical report^18^, which uses only CNN for detecting isotopes and heuristics for grouping the isotopes into peptide features. On the other hand, the new model offered in this paper uses two separate deep learning module IsoDetecting and IsoGrouping, both developed by combining CNN and RNN without using any heuristics.

Traditional methods of detecting peptide features from LC-MS map apply different heuristics and none of them relies on deep learning to find out the appropriate parameters automatically from the available LC-MS data. MSight^19^ generates images from the raw MS data file for adapting the image-based peak detection. CentWave^20^ identifies interesting centroids and then the centroids are collapsed into a one-dimensional chromatogram, and wavelet-based curve fitting is performed to separate closely eluting signals. In MaxQuant^21^, peaks (isotopic signals) are detected by fitting a Gaussian peak shape, and then the peptide feature is found by employing a graph theoretical data structure. AB3D^5^ first roughly picks all local maxima peaks whose intensity is larger than a given threshold, then applies an iterative algorithm to process neighboring peaks of each to form peptide feature. TracMass^22^ and Massifquant^23^ use a 2D Kalman Filter (KF) to find peaks in highly complex samples. Dinosaur is proposed by Teleman *et al*.^24^ where the workflow of feature finding involves centroiding on LC-MS map, assembling centroid peaks into single isotope traces (hills), clustering of hills by theoretically possible *m*/*z* differences, and finally deconvolution of clusters into charge-state-consistent features. Evaluation of peptide feature detection algorithms is challenging because manual annotation of peptide features is out of scope due to the huge size of the LC-MS maps^20^. As a result, most of the literature mentioned above prepare the ground truth data by either taking a common set of peptide features generated by multiple algorithms or a list of MS/MS identified peptides. Some literature treat the ground truth data as true positives, and detection outside those ground truth data as false positives, thus report performance in terms of several statistical measures, e.g., sensitivity, specificity, etc. For instance, CentWave^20^ provides high sensitivity with high precision. AB3D^5^ gives good sensitivity but poor precision. Massifquant^23^ provides high sensitivity with high specificity. On the other hand, there are arguments supporting that we cannot label the peptide features as true positives or false positives in a LC-MS map perfectly. Because a multi-isotope pattern in LC-MS map that is not detected as peptide feature, nor identified later by peptide identification tools, might actually be a peptide feature or merely a noise. We are not definite about their existence since no peptide feature detection tool or identification tool is perfect. Therefore, the percentage of MS/MS identified peptides matched with the peptide feature list produced by different algorithms is used for performance evaluation. For instance, Dinosaur^24^ reports higher matching with MS/MS identified peptides than other existing tools.

In most of the existing algorithms for peptide feature detection, many parameters are set based on experience with empirical experiments, whose different settings may have a large impact on the outcomes. In contrast to these existing works, our research aims at systematically training a deep neural network using real dataset to automatically learn all characteristics of the data, without human intervention. Last but not least, even if the model makes wrong predictions, the correct results can be put back as new training data so that the model can learn from its own mistakes. We believe that such models shall have superior performance over existing techniques and shall become the method of choice soon.

## Results

**Figure 1:**
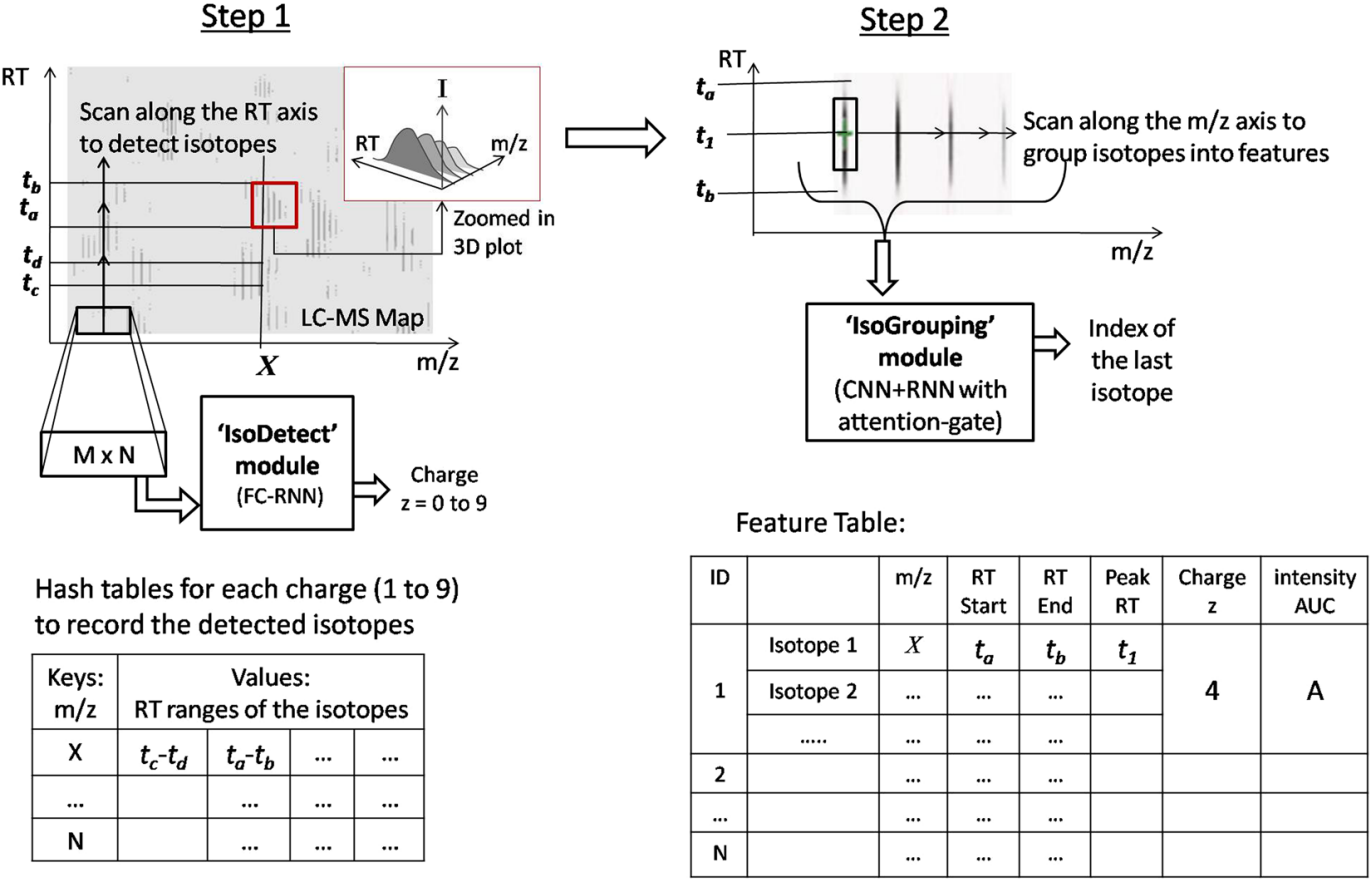
Workflow of our proposed method to detect peptide features from LC-MS map of protein sample.

We explain the intuition of our proposed model using the workflow shown in Fig. 1. It consists of two steps and works on raw LC-MS maps without any preprocessing for noise removal. In the first step, the IsoDetecting module scans the LC-MS map along the RT axis to detect the isotopes having the potential of forming features. The scanning window is large enough to see the pattern of the isotopes and determine their charge states (1 to 9) as well. The isotopes are recorded in a hash table. In the second step, the IsoGrouping module goes to the region of detected isotopes and slides another scanning window along *m*/*z* axis to determine the starting and ending isotopes of a feature. Thus it produces a feature table that reports the detected features along with the *m*/*z* of monoisotope (the first isotope of a feature), charge, RT range of each isotope, and intensity.

In the first step, our job of scanning the LC-MS map along the RT axis resembles the video clip classification, where the RT axis is the time horizon. Therefore, we build IsoDetecting module combining CNN and RNN in a FC-RNN fashion proposed by Yang *et al*.^16^, which achieved state-of-the-art results in the context of video classification on two benchmark datasets. In the second step, we develop the IsoGrouping module combining CNN with the attention-gated RNN proposed by Pei *et al*.^17^. We use attention gate in this module to concentrate more attention on the frame holding monoisotopes (first isotope in a peptide feature) while grouping the isotopes into peptide features. The IsoDetecting and IsoGrouping modules are trained separately using suitable training data. In the Method section, we elaborate each of the steps in detail along with the training procedure.

We downloaded the benchmark dataset from ProteomeXchange (PXD001091) which was prepared by Chawade *et al*.^25^ for data-dependent acquisition (DDA). The samples consist of a long-range dilution series of synthetic peptides (115 peptides from potato and 158 peptides from human) spiked in a background of stable and nonvariable peptides, obtained from *Streptococcus pyogenes* strain SF370^26^. Synthetic peptides were spiked into the background at 12 different concentration points resulting 12 samples each having multiple replicates. We obtain LC-MS map from each replicate, totaling 57 LC-MS maps for the experiment. We cut peptide features from these maps for model training. We apply *k* = 3 fold cross validation^27^ technique to evaluate our proposed model. In each fold, we use 12 maps for model training, 4 maps for model validation, and 41 maps for model testing. Model validation step is a part of training that is used to choose the best state of the model. In the following sections, we will first elaborate the training and validation sensitivity of the model. Then we will evaluate the performance of DeepIso by comparing it with existing tools. The ground truth is set differently for training and performance evaluation (testing) which are briefly mentioned in the following sections and justified in detail in Supplementary Note C.

### Training of DeepIso

Since CNN and RNN are supervised learning approaches, we need labeled data for training. Human annotation of peptide features is out of scope due to gigapixel size of the LC-MS maps^20^. Therefore, we run the feature detection algorithm of MaxQuant 1.6.3.3 and Dinosaur 1.1.3 on the LC-MS maps and then take the common set of feature lists generated by these two algorithms with a tolerance of 10 ppm *m*/*z* and 0.03 minute RT, as labeled samples for training and validation^14,20,28^. The total amount of samples collected in this way from each charge state is presented in Table 1.

**Table 1:** Class distribution of samples in our dataset consisting of 57 LC-MS maps.

Class (charge state)	1	2	3	4	5	6	7	8	9
Amount	163,038	863,050	428,909	29,183	1,503	653	179	236	233

First, we train the IsoDetecting module that tries to maximize the class sensitivity on validation dataset. Here the class sensitivity is the percentage of samples detected correctly from each class, where classes belong to charge states *z* = 0 to 9. The charge state *z* = 0 represents the absence of features. The sensitivity of this class indicates how well the model distinguishes actual features from noisy traces and separates the closely residing features as well. Because of inadequate training data for features with charge states 6 to 9 as presented in Table 1, we had to apply data oversampling and augmentation in order to increase training samples from these classes. The average sensitivity and precision of the trained model on training set and validation set are provided in Table 2. Due to the lack of variance in training data for charge states 6 to 9, the model’s validation sensitivity does not reach close to 90% for these classes. However, since most of the peptide features appear with charge states <6, lower sensitivity for them does not impact the overall performance.

**Table 2:** Class sensitivity and precision of IsoDetecting module and amount of samples for training and validation.

Class (*z*)	Training			Validation		
	Dataset Size	Sensitivity (%)	Precision (%)	Dataset size	Sensitivity (%)	Precision (%)
0	257,250	98.83	99.73	28,992	97.62	99.21
1	21,345	98.19	96.47	3126	94.53	96.23
2	131,951	98.94	96.94	26,480	98.18	95.91
3	59,045	99.26	95.95	10,903	97.95	94.12
4	6,765	99.38	96.07	646	95.72	92.23
5	4,140	98.28	97.36	20	86.59	82.32
6	8,446	99.91	96.48	30	40.36	18.04
7	3,324	94.28	97.87	10	50.00	16.00
8	4,060	99.66	97.44	15	61.79	61.80
9	4,203	99.69	96.58	14	28.21	76.74

Training set for class 5 has some duplicated samples (oversampling). Training sets for class 6 to 9 have both augmented and duplicated samples. Amount of samples from class 0 depends on our choice (discussed in Method section) and this is kept higher than other classes because the LC-MS map is very sparse. The validation set does not contain any duplicated data and there is no overlapping between validation dataset and training dataset.

In the second step, the sensitivity of IsoGrouping module is defined as the percentage of features reported with the correct number of isotopes. The training samples were distributed in five classes denoted as A, B, C, D, and E. Class A associates with noisy traces which does not form any feature. Class B, C, D, and E correspond to features with 2, 3, 4, and 5 isotopes (to be a feature it must have at least two isotopes). Since the scanning window slides left to right, it can handle the cases when peptide features have isotopes over five (details are provided in Method section). We see the training and validation sensitivity in Table 3. We observe that the sensitivity of most of the classes is below 80%. To have a better perception we present the confusion matrix in Table 4. We see the model hardly misses the monoisotopes, but confuses about the last isotope of a peptide feature. Please note that reporting the monoisotope along with first few isotopes (having higher intensity peaks) of a feature is more important in the workflow. Because they dominate the feature intensity and used in the next steps of protein quantification and identification. Therefore we accept a feature if the monoisotope along with high intensity isotopes are reported correctly. Then we choose the state of IsoGrouping module that maximizes the percentage of feature-matched MS/MS identifications on validation dataset.

**Table 3:** Class sensitivity of IsoGrouping module on training set and validation set.

Class	Sensitivity on Training Set (%)	Sensitivity on Validation Set (%)
A (noise)	95.06	94.68
B (2 isotopes)	56.49	57.52
C (3 isotopes)	72.24	72.41
D (4 isotopes)	72.69	74.23
E (5 isotopes or more)	72.41	72.67

**Table 4:** Confusion matrix produced by IsoGrouping module on validation dataset.

Class	A	B	C	D	E
A	94.68%	2.77%	1.73%	0.57%	0.25%
B	3.4%	57.52%	33.86%	4.59%	0.62%
C	0.89%	5.59%	72.41%	20.19%	0.93%
D	0.31%	0.89%	16.18%	74.23%	8.39%
E	0.79%	0.37%	2.70%	23.46%	72.67%

The diagonal values, e.g. [C, C] represent the sensitivity for class C. We say a feature is misclassified as class A when the monoisotope (first isotope) or all of the isotopes are missed, i.e., the feature is thought to be noise by mistake. The value of [C, A] indicates what percentage of features with three isotopes are either misclassified as noise, or monoisotope is missed. [C, B] indicates the percentage of features which actually have three isotopes but the third one is missed, and only first two are combined together. Similarly [C, D] shows the percentage of three isotope features, for whom IsoGrouping module finds ONE additional isotope at the end.

### Performance Evaluation of DeepIso

For performance evaluation, we present the percentage of high confidence (i.e., high quality) MS/MS peptide identifications matched with the peptide feature list produced by our algorithm. Since the identified peptides must exist in LC-MS maps, therefore, the more we detect features corresponding to them, the higher the performance^5,14,20,28^. We run MASCOT 2.5.1 to generate the list of MS/MS identified peptides and the identifications with peptide score >25 (ranges approximately from 0.01 to 150) are considered as high confidence identifications^5^.

In this testing phase we first scan the LC-MS map by IsoDetecting module. Then another run of scan by IsoGrouping module goes through the potential patterns detected in the first step, and reports the final list of peptide features. In order to compare performance of our model with other existing tools, we run the peptide feature detection algorithm of MaxQuant 1.6.3.3, OpenMS 2.4.0, and Dinosaur 1.1.3 as well. We used the published parameter for MaxQuant as reported by Chawade *et al*.^25^. For Dinosaur, default parameters mentioned at their github repository (https://github.com/fickludd/dinosaur) are used. For OpenMS, we use the python binding pyOpenMS^28,29^ and follow the centroided technique explained in the documentation (https://pyopenms.readthedocs.io/en/latest/feature_detection.html). For all of the feature detection algorithms, we set the range of charge state 1 to 9 (or the maximum charge supported by the tool). Then the produced feature lists are matched with the high confidence MS/MS identifications with tolerance of 0.01 *m*/*z* and 0.2 minute RT.

As mentioned earlier, we have 12 samples where sample 2, 3, and 4 have 7 replicates each and the remaining samples have 4 replicates each. We show the average percentage of high confidence MS/MS identifications matched with the detected peptide features for 12 samples in Table 5 (entire result can be found in Supplementary Table S3).

**Table 5:** Percentage of high confidence MS/MS identifications matched by feature list produced by different algorithms.

Algorithms	MaxQuant	OpenMS	Dinosaur	DeepIso
Matching	96.83%	97.14%	97.23%	97.43%

Although the performances are quite close, however, DeepIso is still a little bit ahead of all others which indicates that deep learning tools are desired to advance the state of the art techniques. It is able to report some features not detected by other tools as provided in the Venn diagram of feature-matched MS/MS identification by different tools in Supplementary Fig. S2. In Discussion section we explain the scenario when our model might miss a feature, and propose potential solution to overcome the problem, thus increasing the sensitivity further as well.

**Figure 2:**
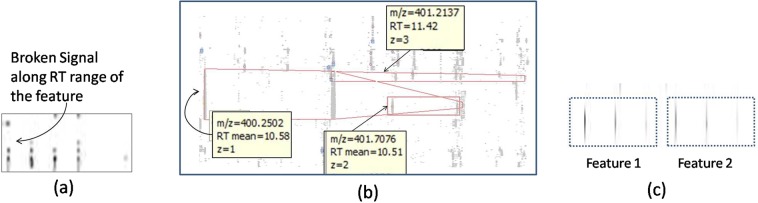
(**a**) A peptide feature with broken signals; (**b**) Detection of overlapping peptide features; (**c**) Adjacent feature case.

We would like to mention how the retraining with misclassified cases promotes better learning in our model. As mentioned earlier, if a deep learning model does mistakes, we can collect those cases and retrain the model with those specific cases which improves the model’s ability in giving the correct result next time. We applied this retraining process while building the IsoGrouping module. The module was unable to separate adjacent features, e.g., feature 1 and 2, shown in Fig. 2(c). We collected such cases and retrained the model which improved the overall matching by about 4% (details are provided in Supplementary Note B). Therefore, such model can evolve as new cases appear.

#### Peptide Feature Intensity Calculation by DeepIso

Next would like to verify the correctness of peptide feature intensity calculation by our model. For the statistical analysis of biological experiments the peptide feature intensity is of interest and has to be calculated from raw data^20^. The technique is to first apply curve fitting over the bell shaped intensity signals of isotopes in a feature. Then the Area Under Curve (AUC) of all isotopes in a feature are calculated and added to get the intensity or AUC of that feature. Therefore the perfectness of peptide feature intensity depends on whether the bell shaped signals are detected nicely or not. We report the Pearson correlation coefficient of the peptide feature intensity between DeepIso and other existing algorithms in Table 6. It appears that our algorithm has a good linear correlation with other existing algorithms, which validates the peptide feature intensity calculation by our model.

**Table 6:** Pearson correlation coefficient of the peptide feature intensity between DeepIso and other tools.

	Dinosaur	MaxQuant	OpenMS
**DeepIso**	87.73%	88.99%	91.46%

#### Time Requirement of DeepIso

Total time of scanning LC-MS map by IsoDetecting module and IsoGrouping module is considered as the running time of DeepIso model. The running time of different algorithms along with the platforms used in our experiment is presented in Table 7. It appears that our DeepIso model has a higher running time than Dinosaur and Maxquant. However, this running time can be improved if we increase the available GPU for parallel processing. We also propose some potential methods to speed up DeepIso model in Discussion section.

**Table 7:** Approximated running time of different algorithms.

Platform	Processor: Intel Core i7, 4 cores OS: Windows 10 for running the applications		Processor: Intel(R) Xeon(R) Gold 6134 CPU, NVIDIA Tesla OS: Ubuntu 16.04.5 LTS for running the python scripts	
Algorithms	Dinosaur	MaxQuant	**DeepIso**	OpenMS
Running Time	15 minutes	30 minutes	**1 hour and 40 minutes**	2 hours and 50 minutes

Here the platform used for OpenMS and DeepIso did not have support for running Windows application of MaxQuant and Dinosaur. So we used different machine for running those.

Finally, we would like to state that, although the common set of Dinosaur and MaxQuant was used to prepare training data, it does not imply that our model just learns to mimic their approach. We used the common set merely to replace human annotators to label the training data. DeepIso learns the appropriate parameters for peptide feature detection by stochastic gradient descent through several layers of neurons and backpropagating the prediction errors, a completely different approach than existing heuristic methods. Therefore the learning outcome of this deep learning model is quite different. Moreover, once we train a model on a protein sample, the same model should be applicable to all other protein samples from the same or other close species without further training. We believe our empirical results reveal the capability of deep learning networks in solving peptide feature detection problem, as well as, discloses the area of improvements to make it more robust and high performing in the future.

## Discussion

We propose DeepIso, a peptide feature detection algorithm that does not apply human designed heuristics involving centroiding, curve fitting, clustering, etc. Rather, it uses the power of deep neural networks to automate the learning of peptide feature detection by revealing the important feature characteristics from LC-MS data. We will first demonstrate the justification of different design strategies followed and the utility of our model from industrial perspective. Then we will discuss the shortages of the current model and propose potential solutions to overcome the problems.

We would like to explain the significance of using RNN along with CNN for peptide feature detection. In the initial stage of this research we used only CNN in IsoDetecting module and units were set to be 0.01 minute along RT axis. Only about 73% of the MS/MS identified features are reported in that technique, whereas, about 97% are reported in current model due to using RNN along RT axis. The RNN cells in the IsoDetecting module helps to detect the features having broken signals as shown in Fig. 2(a). Besides that, using MS-Scans as units of RT axis let us avoid human intervention caused by interpolating signals at the missing MS-Scans on some units of RT axis. It also makes the whole scanning faster and prevents the network from confusing broken features with the noisy traces. Please see Supplementary Table S2 for the experimental details.

Now we discuss about the reason of using simple RNN cells in IsoDetecting, instead of Long Short-term Memory (LSTM)^30^ cells. Although the span of LC-MS map along RT axis is very long, but the RNN does not need to look back very far in the past to detect an isotope, since each isotope’s RT range is not very long. After start detecting a feature (charge *z* with value 1 to 9), it has to remember the states upto the end of the feature (*z* = 0) only. After that it can refresh its memory. This is why we did not use LSTM cells to make the network unnecessarily complicated.

We do not use any pooling layers in the network of IsoDetecting module. In order to detect the sharp boundary and location of the peptide features, we want the network to have the property ‘equivariant to translation’ (ensured by CNN filters) to generalize edge, texture, shape detection in different locations, but not ‘invariant to translation’ (ensured by pooling layers) that causes the *precise location* of the detected features to matter *less*, and give unexpectedly wider detection for isotopes (see Supplementary Fig. S5). So we avoid using pooling layer.

In IsoDetecting module the frame size of [15 × 211] (covering 15 scans and 2.11 *m*/*z*) ensures that it sees reasonable area of a feature to decide about the existence of it along with the charge. If we reduce the frame size then we have to use two dimensional and bi-directional RNN in IsoDetecting module (in order to look at surrounding area). It prevents batch processing of multiple regions of LC-MS map making the whole process time consuming. Smaller frame size might also hamper the power of pattern detection by CNN.

If we use attention-gated RNN in IsoDetecting module then it results in lower class sensitivity as apparent from Table 8. Therefore we chose FC-RNN network for designing this module.

**Table 8:** IsoDetecting module give better validation sensitivity with FC-RNN network than attention-gated RNN.

Class (*z*)	0	1	2	3	4	5
FC-RNN network (better)	96.43%	93.80%	96.98%	98.74%	97.94%	85.86%
CNN with attention-gated RNN	96.15%	89.00%	96.04%	96.46%	95.07%	54.29%

So far we have discussed the methodology for training ‘IsoDetecting’ module. Now we will discuss the same for IsoGrouping module, the second step in our DeepIso model. We present a brief progression of IsoGrouping module that we have gone through to achieve the current state of the model in Table 9. The first row shows the initial IsoGrouping module, designed using FC-RNN network with three convolution layers, one fully connected layer, without any pooling layer and state size 4. It reports peptide features for only about 87% of the MS/MS identified peptides. Then we retrain the model using the aforementioned adjacent feature cases which improves the match drastically by about 4%. Addition of max-pooling layer, more fully connected layers and increased state size gradually improve the performance further. Later we see the network gives better performance with attention-gated RNN instead of FC-RNN. We generate the final result by ensemble of multiple IsoGrouping modules, who differ in terms of initial weights, learning rates, and number of neurons in fully connected layers. We have included the details of each stage in Supplementary Note D.

**Table 9:** Performance of IsoGrouping module in different stages of the development (based on validation dataset).

Model	Matching with MS/MS identified peptides
Initial model	87.55%
Retraining using Adjacent Feature cases	92.82%
Addition of max-pooling layers, one more fully connected layers and state size raised to 8	94.66%
Network with attention-gated RNN	95.08%
Same as above but trained with more data	95.13%
Ensemble of multiple instance of the model	95.46%

Finally, we improve peptide feature detection for charge states 6 to 9 as presented in Table 10. This mostly involves IsoDetecting module. Since the original amount of samples from these classes were negligible according to Table 1, we had to apply data oversampling (equivalent to applying more penalty for misclassifying samples from lower abundant classes) and augmentation (Supplementary Note A for details) to train the module on these classes. It improves the final matching with MS/MS identification as presented in Result section.

**Table 10:** Improvement of class sensitivity of IsoDetecting module for charge states 6 to 9 with increasing amount of training samples.

Class (*z*)	Initial Dataset		Oversampling was performed by duplicating training samples (*z* = 6 to 9) 10 times		Augmented samples were created from training samples (*z* = 6 to 9) and then duplicated 10 times	
	Validation Sensitivity	Training Sensitivity	Validation Sensitivity	Training Sensitivity	Validation Sensitivity	Training Sensitivity
6	0%	0%	52.65%	98.32%	40.36%	99.9%
7	0%	0%	0	96.53%	50%	94.1%
8	0%	0%	31.67%	99.14%	61.80%	99.6%
9	0%	0%	38.57%	98.12%	28.20%	99.7%
Comments	Network does not learn anything due to negligible amount of original samples.		Although the network starts learning because of introducing higher penalty for lower abundant samples, however it still does not learn well due to lack of variance in samples. Network also overfits due to lack of data.		Various samples were created using augmentation which improves the validation sensitivity further. However, the network still overfits due to lack of data amount and variation.	

We do not bother for further improvement (by including more data from different but similar dataset) since most of the peptide features generally appear in LC-MS map with charge states <6. Here the validation set does not contain duplicated data and there is no overlapping among the training set and validation set.

From the industrial perspective it is important to detect peptide features with high intensity signals properly. To compare this property of different algorithms, we sort the peptide feature list generated by different algorithms based on ascending order of peptide feature intensity. Then we select the top 10,000 peptide features (about 20% of the existing peptide features in each LC-MS map, which is about 50,000) from each list and denote them as high confidence feature list. Finally we compare that list with the high confidence MS/MS identifications. DeepIso provides 89.32% matching which is higher than Dinosaur (89.24%), MaxQuant (87.65%), and OpenMS (60.44%). The performance of OpenMS is lower than others, because it produces some high intensity false positives. We believe, the good performance by DeepIso makes it a suitable model for industrial sector as well.

**Figure 3:**
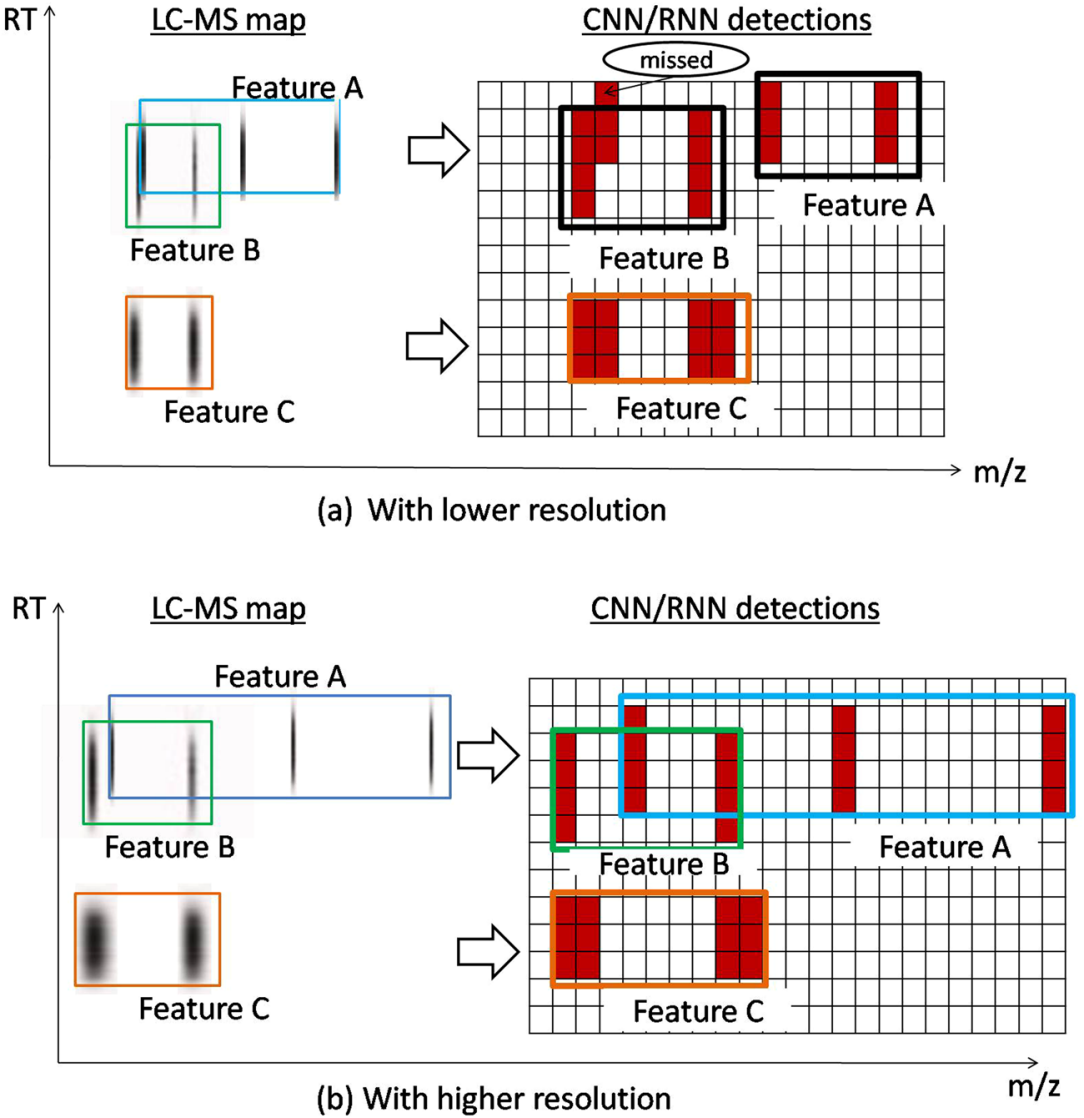
Intuitive image showing the effect of resolution along the *m*/*z* axis of LC-MS map: (**a**) lower resolution merges the closely residing peptide features; (**b**) higher resolution separates the same feature and let the IsoDetecting module perform correct detection.

Now, we would like to mention some disadvantages in our proposed DeepIso model. Visual observations at some peptide features on LC-MS map discover that some features are missed due to the lower *resolution* considered for *m*/*z* axis. Although we are able to teach DeepIso to detect overlapping features as shown in Fig. 2(b), detection of some closely residing peptide features (with close monoisotopic peaks) in the LC-MS map, for instance, feature A and feature B in Fig. 3(a), are merged together. Since we do not want to use any heuristics on them, we are unable to separate such detection associated with A and B. However, if we increase the resolution as shown in Fig. 3(b), then the features are separated in LC-MS map and thus isolated by IsoDetecting module as well. Therefore, increasing the resolution will let IsoDetecting module see the LC-MS map better and result in higher performance. However, in that case we will need to sacrifice the time efficiency since increasing resolution by one decimal point, for instance 0.01 to 0.001 will make the input frames 10 times bigger in dimension and eventually resulting in larger feature maps, turning the model slower than before. Therefore we have to find an intelligent architecture that will let us increase the resolution without compromising running time.

Finally, we would conclude by proposing some potential future scopes. We believe there is still room for improvement in peptide feature detection context mostly owing to the overlapping peptides and the multi-isotope patterns that sometimes defy typical assumptions of peptide features. Deep learning networks are desirable for the ability of self learning from training data without human interventions. However, time efficiency is also an important factor considering the practical utility. The running time of DeepIso is dominated by the first step, IsoDetecting module. Because it has to scan the whole LC-MS map represented as a 2D image of gigapixel size (about [12,000 × 140,000] pixels). Therefore one of our next concern is to make the IsoDetecting module time efficient. At the same time we would like to consider increased resolution along the *m*/*z* horizon of LC-MS maps to improve the feature detection, as mentioned lately. One potential approach might be using PointNet^31^, which avoids 2D image representations of 3D objects, and directly works on the point cloud (set of data points in space). In addition to that, designing the IsoDetecting module as a segmentation network might be helpful as well. Besides that, whether using ‘BERT’^32^ technique (recent advancement in the context of natural language processing) in implementing IsoGrouping module brings better performance is also left for future research. Application of DeepIso in Label Free Quantification (LFQ) can be another direction of work. We are looking forward to these research opportunities in the future.

## Methods

Our model runs the processing on raw LC-MS map. We use the ProteoWizerd 3.0.18171^33^ in order to obtain the ‘.ms1’ format of the raw LC-MS maps. Then we read the file and convert it to a 2D grey scale image (i.e. RT × *m*/*z* plot) by treating the third dimension ‘Intensity’ as a grey value scaled between 0 to 255.

### Step 1: Scanning of LC-MS map by IsoDetecting module to detect isotopes

**Figure 4:**
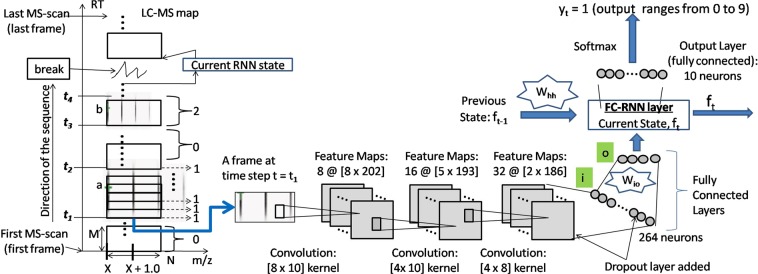
Network of IsoDetecting module.

According to our design, this step is basically a 10 category classification problem. Please refer to the LC-MS map represented as a *RT* × *m*/*z* plot shown in Fig. 4 for the clarification on the scanning process. Our network scans the LC-MS map as a sequence of [*M* × *N*] dimension frames, where each sequence is positioned at a point on the *m*/*z* axis (for instance *X*) and the time steps range from the first MS-scan to the last MS-scan along the RT axis. We name a scanning through each sequence like this as one round of ‘deep scan’. In this figure, we see a sequence passing over the isotopes of two features, feature ‘a’ and ‘b’ having charge ‘1’ and ‘2’ respectively. At each time step, the network outputs one of the classes in the range 0 to 9, 0 being the class indicating ‘No Feature Seen’, and 1 to 9 being the classes indicating features seen having corresponding charges. For instance, in the figure we use some dotted arrows to indicate the network outputs (charge 1) at the corresponding time steps. The network outputs 0 in the blank spaces or noisy traces. Please note that, the scanning window, that is the frame has dimension [*M* × *N*] = [15 × 211] which is large enough to see the second isotope (along the *m*/*z* axis) in a potential feature to take decision about the charges. We do this to avoid using bidirectional RNN. The frames are overlapping as well so that we can trace the RT range of the isotopes nicely.

Each unit of RT axis represents one MS-scan (who are at least 0.01 minute apart) and each unit of *m*/*z* axis equals to 0.01 *m*/*z*. However, each MS-scan does not hold signals from all m/z points. Therefore there are breaks in a sequence of ‘deep scan’ as shown in the LC-MS map (in Fig. 4) by a broken line, where we simply pass the current RNN state to the next available frame. Please note that, the states of one ‘deep scan’ are passed along the RT axis. Therefore, one ‘deep scan’ positioned at *X m*/*z* is independent of another ‘deep scan’ positioned at *X* + 0.01 *m*/*z* and vice versa. So we can process multiple ‘deep scan’s in a batch which makes the whole approach time efficient.

We keep nine hash tables for recording the detection coordinates (the points in *RT* × *m*/*z* plot) of features from nine classes (*z* = 1 to 9) during the ‘deep scan’. The *m*/*z* values of the isotopes are used as the key of these hash tables, and the RT ranges of the isotopes in a feature are inserted as values under these keys as shown in the block diagram of Fig. 1. Since the detection of wider isotopes may span over a range of *m*/*z* (i.e., multiple pixels along *m*/*z* axis as shown for feature ‘C’ in Fig. 3), we take their weighted average to select specific *m*/*z* of a isotope.

The deep learning network is shown in Fig. 4. The network is taking the frame at time step *t* = *t*_1_ as input. There are three convolution layers, followed by two fully connected layers (denoted as *i* and *o*), one FC-RNN layer, and output is generated at each time step. The dropout layer is added after third convolution layer and first fully connected layer *i* with a value of 0.50, which is considered ideal for many cases. We use state size 4 and the tanh activation function. Since we are dealing with FC-RNN model^34^, the state *f*_*t*_ at time step *t* is defined as below:

1 $${f}_{t}=H({W}_{io}.{X}_{it}+{W}_{hh}.{f}_{t-1}+{b}_{o})$$

Where in, *H* is the activation function, *W*_*io*_ is the weight matrix connecting the neurons of layer *i* to layer *o* (as shown in Fig. 4), *X*_*it*_ is the output of the layer *i* at current time step *t*, *W*_*hh*_ is the weight matrix of RNN state, *b*_*o*_ is the bias at layer *o*, and *f*_*t*−1_ is the previous state.

#### Training Procedure of IsoDetecting module

Now we will discuss about the training procedure of the network. It is supposed to learn following basic properties of peptide feature^35^, besides many other hidden characteristics from the training data.

1. In the LC-MS map, the isotopes in a peptide feature are equidistant along *m*/*z* axis. For charge *z* = 1 to 9, the isotopes are respectively 1.00 *m*/*z*, 0.5 *m*/*z*, 0.33 *m*/*z*, 0.25 *m*/*z*, 0.17 *m*/*z*, 0.14 *m*/*z*, 0.13 *m*/*z*, and 0.19 *m*/*z* distance apart from each other^12^.
2. The intensities of the isotopes form bell shape within their retention time (RT) range as shown in the zoomed in view of Fig. 1.
3. Peptide features often overlap with each other.

Training sequences are 20 frames long. Positive samples are created by cutting a sequence that is aligned with the monoisotope of the peptide features. We cut sequences from blank or noisy areas, not aligned with any feature and treat them as negative samples. In this way we generate approximately 200,000 positive samples and 200,000 negative samples. Since the IsoDetecting network produces output at each time step, we label each frame of a sequence with one of the classes ranging from 0 to 9. We deal with variable length sequences since the peptide features might not span over 20 frames (scans). We apply data augmentation for training samples having charge states 6 to 9. Details on training data generation are provided in Supplementary Note A. We use ‘Adam’ stochastic optimization^36^ with initial learning rate of 0.01. We use sparse softmax cross entropy as error function at the output layer. We run about 100 epochs and the model starts converging after about 90 epochs.

### Intermediate Step to Make Clusters

**Figure 5:**
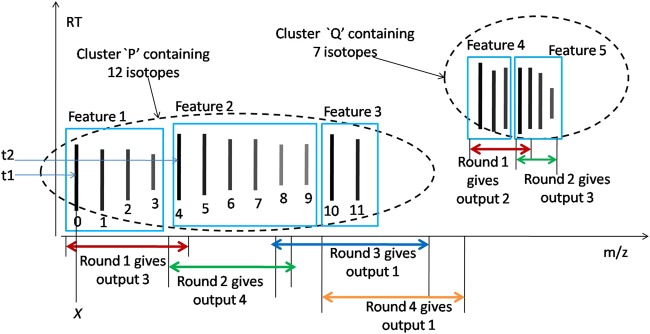
Intuition of scanning by IsoGrouping module on the clusters of features.

We use an intermediate step that forms clusters of closely residing isotopes having same charge, overlapping RT extent, and equidistant along the m/z axis. In other words, the equidistant isotopes of same hash table are grouped into one cluster. For instance, we see two clusters ‘P’ and ‘Q’ in Fig. 5. Look that, same cluster might hold multiple peptide features. This step is designed just to speed up the whole process by allowing batch processing in the second step. Each batch containing about 500 clusters are passed to the IsoGrouping module. Detecting the starting and ending of features in the clusters is handled by this module. Please note that, this step is optional and avoiding this step does not bring any significant change in the result. However, the running time of IsoGrouping module increases drastically due to not utilizing the power of batch processing. We do not set any limit on the maximum number of isotopes per cluster. Experiment shows that each cluster usually holds at most 16–20 isotopes.

### Step 2: Scanning of LC-MS Map by IsoGrouping Module to Report Peptide Feature

In this step the frames are placed at the isotopes of the clusters. For convenience please refer to Fig. 5. There are two major differences in IsoDetecting and IsoGrouping modules. First, the IsoDetecting module scans the LC-MS map along the RT axis, whereas IsoGrouping module scans left to right, along *m*/*z* axis. Therefore, the time steps span along the m/z axis. Second, IsoGrouping module generates one output after seeing through ‘5’ consecutive frames (after 5 time steps), unlike IsoDetecting module which generates output at each time step. Here, each cluster is processed in multiple rounds. Starting frame of one round depends on the output of previous round and the rounds can be overlapping as well. A step by step explanation of the scanning procedure with figure is provided in Supplementary Method A.

**Figure 6:**
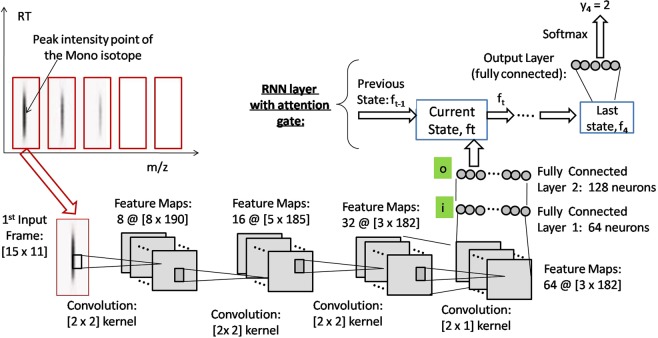
Network of IsoGrouping module.

The network is shown in Fig. 6. It has four convolution layers, followed by two fully connected layers. This time we include pooling layers after first and second convolution layers. The dropout layers are included after each fully connected layers with dropout probability of 0.5. We input the charge *z* detected by the IsoDetecting module as a feature at the layer *i* as shown in the figure. We do this by concatenating *z* with the output *X*_*i*_ of layer *i*. We use state size 8 and tanh activation function. The current state *f*_*t*_ at time step *t* is calculated using attention gate *a*_*t*_^15^ as follows:

2 $${f}_{t}=(1-{a}_{t}).{f}_{t-1}+{a}_{t}.{f^{\prime} }_{t}$$

wherein, *f*_*t*−1_ is the previous hidden state, *f*′_*t*_ is the current state calculated in the conventional fashion and *a*_*t*_ denotes the importance of current frame to the final decision. The *f*′_*t*_ and *a*_*t*_ are calculated as below:

3 $${f^{\prime} }_{t}=H({W}_{hh}.{f}_{t-1}+{W}_{oh}.{X}_{ot}+{b}_{h})$$

4 $${a}_{t}=\sigma ({W}_{a}.{f^{\prime} }_{t}+{b}_{a})$$

In Eq. 3, *H* is the activation function, *W*_*hh*_ is the weight matrix connecting the previous hidden state *f*_*t*−1_ to the current state, *X*_*ot*_ is the output of the layer *o*, *W*_*oh*_ is the weight matrix connecting the *X*_*ot*_ to the RNN layer, *b*_*h*_ is the bias at the RNN layer. In Eq. 4, *σ* is the sigmoid activation function, *W*_*a*_ is the weight matrix that learns the attention mechanism and *b*_*a*_ is the corresponding bias.

#### Training Procedure of IsoGrouping module

Usually monoisotope’s intensity is the highest among the other isotopes in a feature and dominates the total intensity of the feature. This property should be learnt by IsoGrouping module. We generate the positive samples by generating a sequence of 5 frames for each peptide feature, where the sequence starts at the first isotope of the respective feature. Each frame has dimension [15*x*10], covering 15 scans along RT axis and 10 units along the *m*/*z* axis. The frames are centered on the point associated with the peak intensity of the monoisotope, as shown in Fig. 6. Each sequence is labeled by the frame index holding the last isotope of the feature (indexing starts from 0). If the feature has more than 5 isotopes, than it is labeled as ‘4’. In this way we generate about 220,00 positive samples. We generate negative samples by cutting some sequences from the noisy or blank area. We also generate sequences that contain peptide feature, but the feature does not start at the first frame of the sequence. Those samples are labeled as ‘0’ and considered as negative samples as well. We do this to handle the cases where noisy traces are classified as isotopes by IsoDetecting module by mistake and thus clustered with the actual features in the intermediate step. We generate about 120,000 negative samples. We apply ‘Adagrad’ stochastic optimization^37^ with initial learning rate of 0.07. We use softmax cross entropy as error function at the output layer.

### Ensemble of Multiple IsoGrouping Modules

In order to reduce variance we use ensemble^38^ of multiple IsoGrouping modules to report the peptide features. We generate four instances of the IsoGrouping module, who are different in terms of initial weights, learning rate (0.07, 0.08), state size (6, 8, 10), and size of the second fully connected layer (80, 128). Their outputs are combined using soft voting^39^. Ensemble technique improves the matching with identified peptides by about 0.33%. Please see Supplementary Table S1 for details.

We would like to mention the common strategies followed for implementing and training both of the modules. We implemented our deep learning model using the Google developed Tensorflow library. However, we had to build the RNN network ourselves instead of using their built-in RNN cells, in order to reflect the gating mechanism proposed in FC-RNN^16^ and attention gated RNN cells^17^. During the training of both modules, we use minibatch size of 128 to ensure enough weight update in each epoch. We check the accuracy on validation set after training on every 10 minibatches. We perform data shuffling after each epoch which helps to achieve convergence faster. We continue training until no progress is seen on validation set for about 5 epochs. Including dropout layer in our model increases the validation sensitivity by about 1.5%. Although the Rectifier Linear Unit (ReLu) activation function is preferred over tanh in many literature, our model does not learn well with ReLu according to our experiments.

## Supplementary information

Supplementary Documents

## Data Availability

The benchmark dataset is available to download from ProteomeXchange using accession number PXD001091. The full experimental result on all the replicates of the samples are available in supplementary materials.
