# Supplementary material for: DeepIso: A Deep Learning Model for Peptide Feature Detection from LC-MS map
Source: Sci Rep. 2019 Nov 20;9:17168. doi: 10.1038/s41598-019-52954-4 (PMC6868186; doi:10.1038/s41598-019-52954-4)
Supplement: Supplementary file 1 — Supplementary Documents [file 41598_2019_52954_MOESM1_ESM.pdf]

DeepIso: A Deep Learning Model for Peptide Feature Detection from LC-MS map

Fatema Tuz Zohora, M Ziaur Rahman, Ngoc Hieu Tran, Lei Xin, Baozhen Shan, Ming Li

Supplementary Note A

Training Data Generation for 'IsoDetect' Module

Each training sample is consists of 20 frames. That means it covers 20 consecutive scans (who are at least 0.01 minute apart). Positive samples are created by cutting a sequence that is aligned with the monoisotope of the peptide feature as shown in Supplementary Figure S1. The actual feature boundary is shown using dotted rectangle. The sequence starts 2 scans earlier than the actual start of the feature so that the network learns the gamma shape of intensities nicely. Similarly, the frames are positioned 10 ppm earlier than the given m/z of the monoisotope to make it error tolerable and let it see the whole isotope in case of wider isotopes. However, a peptide feature might span over less than 20 scans and that is why we deal with variable length sequences. We cut sequences from blank or noisy areas not holding features and treat them as negative samples. In this way we generate about 200k positive samples and 200k negative samples. Please note that, our 'IsoDetect' network produces output at each time step. Therefore we label each frame of a sequence with one of the classes ranging from 0 to 9 (as shown 0, 0, 1, ... for the first three frames in the figure).

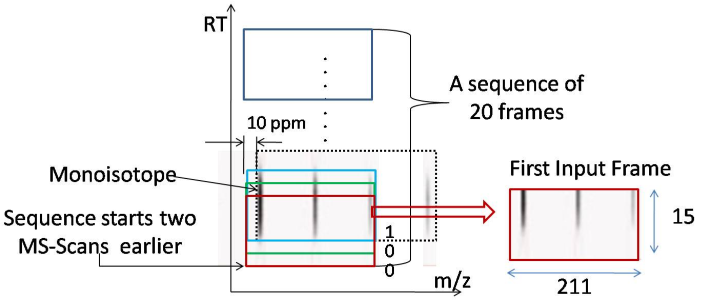

Supplementary Figure S 1. Training data generation for the 'IsoDetect' module

Augmented Data Generation for 'IsoDetect' Module

For charge states 6 to 9, we did not have enough samples for training. Therefore we applied data augmentation. Please note that the 10 ppm tolerance along m/z axis let us cut the features couple of pixels before the exact start, as mentioned above. This number of pixels can vary from 0 to 2 based on the actual m/z. Since the LC-MS maps in our dataset span from 400 m/z to 2000 m/z (approximately), therefore for each sample having these charge states we can **cut multiple sequences** within the tolerance limit. For example, if a peptide feature with charge state 6 lies around 2000 m/z area, then tolerance limit is up to 2 pixels. So we cut sequences starting at exact m/z, 1 pixel (or 0.01 m/z) before, and 2 pixels (0.02 m/z) before. So we get three sequences for it. In this way we generate augmented samples.

Supplementary Note B

Adjacent Feature case

This case appears when two features having same charge state (e.g., z=2) reside one after another such that, distance between the last isotope of the first feature, and the monoisotope (first isotope) of the second feature, is equal to the inner isotope gap of those features (distance between two successive isotopes of the same feature). Please see Figure S 2(a) for clarification. We show two peptide features 'p' and 'q', having same charge and inner isotope gap of 0.50 m/z. This pair of peptide features create a adjacent feature case. Another pair of peptide features holding 'r' and 's' causing the same problem is also shown in this figure. The Adjacency Feature case might involve more than two peptide features in a row.

Misclassification of Adjacent Feature case

We noticed that the isoGrouping module was doing mistakes in separating such adjacent features while sliding the scanning window from left to right over these peptide features. A peptide detection is considered correct if the monoisotope is reported accurately. We redraw the 1st pair, peptide feature 'p' and 'q' as in Figure S 2(b). Here we see how the isoGrouping module sees them during scanning. It just sees a bunch of isotopes, which we index as 0 to 5 for convenience. Following three types of mistakes were observed:

- Isotope 0 to isotope 3, these four isotopes are grouped as peptide feature ‘p’. And then the rest two isotopes 4, and 5 were grouped as peptide feature ‘q’. As a result, peptide feature ‘q’ misses the monoisotope, which is considered as missed feature. Because for a detection to be correct, the monoisotope of the peptide must be accurate.
- Only isotope 0 and isotope 1 are grouped as peptide feature ‘p’. And then from isotope 2 to isotope 5, these 4 isotopes were grouped as peptide feature ‘q’. This time although isotope 3 exists on peptide ‘q’, however the isotope 2 is reported as monoisotope of this peptide. This is also considered as missed feature, since monoisotope is wrong.
- Sometimes the isoGrouping module group all of the isotopes into just one big peptide feature ‘p’. As a result the second peptide ‘q’ is again missed.

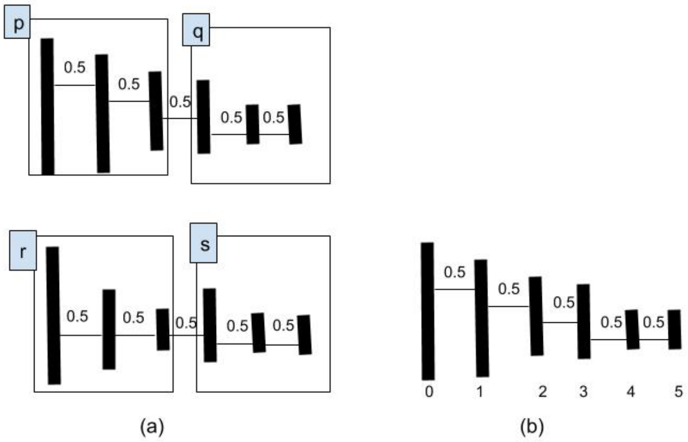

Supplementary Figure S 2. ‘Adjacent Feature’ problem

Retraining to teach Adjacent Feature case

For each fold we do the following steps:

- We select three samples, e.g., 10, 11, 12 for training the IsoGrouping module using the training set prepared as mentioned in Method section. Please recall that the percentage of feature matched MS/MS identification was used to verify the performance of IsoGrouping module. Two LC-MS maps, e.g., 9_01, 9_02 are used for validating the isoGrouping module. We first scan these two LC-MS maps using IsoDetecting module. The produced isotope lists are passed to the IsoGrouping module for validating through matching with MS/MS identifications.
- During training IsoGrouping module, after the model reaches saturation we chose the best state of the model based on MS/MS identifications matched with reported features for 9_01, 9_02. Then we find out the MS/MS identifications from 9_01, 9_02 for which we do not get any match due to adjacent feature case problem. We record that amount as N1 and N2 respectively. The N1 and N2 equal to about 8% of the MS/MS identified peptides.
- Then we do the same scanning (first by isoDetecting and then by isoGrouping) on samples 10, 11, 12 (which were used for training). We see that some of the peptide features are missed as well from these LC-MS maps for adjacent feature case. We compare the feature list generated by DeepIso with the training feature list for these maps (common set of MaxQuant and Dinosaur) and find out about 18,000 adjacent feature cases which were not detected by our model. We cut sequences holding those cases. For example, each sequence would hold two or more peptide features like ‘a’ and ‘b’, as explained in above sections, and also can start from middle isotope of features (initial training sequences for IsoGrouping module consists of one peptide feature only and always start with the first isotope of features). Then we add this set of sequences with the previous training set (duplicated 5 times) and run the training again.

- This time we don't run training from scratch. We saved the model state every 10 epochs while doing the initial training. We choose the state saved at epoch 50, and start retraining from that point. By 90 epochs it approaches to saturation. We also keep track of what percentage of N1 is correctly detected this time. That amount comes close to 97% as the model approaches saturation. Then we choose the best state and save it. We use this retrained model on 9_02 again and see that over 95% of 'N2' are detected this time correctly.

Supplementary Note C

The approach of setting ground truth are different for training and evaluation. We use the common set of two peptide feature detection algorithms as ground truth for model training, because human annotation of dataset is out of scope [20]. This technique of training is also supported by existing research works [14, 20, 25], e.g., the 'retention time prediction' model training in OpenMS. However, we do NOT use that common set for performance evaluation. Rather we use the identified peptide list through database search performed by MASCOT as ground truth. Similar evaluation technique is supported by previous research works [5, 14, 24] as well. Now we will justify our approach first discussing why the usual techniques of ground truth setting for deep learning is not applicable in our case. Then we will show why our training and evaluation technique is appropriate.

Most of the image based object detection algorithms are supervised problems where human annotated datasets are used as training set. The training set generation for such problems is quite easy through crowdsourcing. Even with natural language processing, text annotation is feasible. The news articles, forums, notes from wikipedia and several online articles make it easier to make the training set as big as consisting of millions of labeled samples. There are also several public datasets available for such image or text models.

However, our problem is different from traditional object detection problem in the sense that, here the objects are very tiny in size with respect to the background area, but very big in number. One LC-MS experiment consists of several runs, each producing a two dimensional LC-MS map with size [12,000 x 140,000] pixels (about 12,000 points along Retention time or vertical axis, and more than 140,000 points along m/z or horizontal axis), and each such map containing about 50,000 peptide features. For example, our dataset has 54 such LC-MS maps. To train our model, we need a set of cut peptide features (samples) from each map. However, we do not really know about the actual number of peptide features each map is holding. Because no human being can go through each point of this huge gigapixel image and apply theoretical rules to find multi-isotopic pattern perfectly. Even human effort might produce erroneous results. Therefore the traditional and straightforward technique of using human annotated samples for model training does not work with us. Therefore the main question is how do we fix a ground truth for peptide features to train and evaluate our model?

- To evaluate the performance of our model we see what percentage of MS/MS identified peptides (by database search) are matched with our peptide feature list. The protein identification and quantification workflow is denoted as tandem LC-MS/MS. It starts with analysing LC-MS maps for peptide features. And then the MS/MS spectra are analysed for peptide identification. We have no doubt about the existence of peptide features in LC-MS map which correspond to the identified peptides from MS/MS spectra. The more we detect features corresponding to them, the higher is performance. We further add that, during evaluation we actually select high confidence peptide identifications. A peptide score is assigned to each identified peptide which indicates how confidently that peptide was identified. In our dataset the peptide score ranges from 0.01 to 150 approximately. Based on existing literature we denote the identifications with peptide score > 25 as the high confidence peptides [5].

- Now the second question is how do we train our model. The easiest and most reliable technique would be to use the features matched with database identified peptides (mentioned in previous point) for training as well. However, database search can identify only few thousands of peptides from each map. But we need millions of samples for training. Therefore although database identified peptides are good for evaluation, but not sufficient for training a deep learning model. At this point one big question is, does the rest of 50,000 peptides which are not identified by database search are all false? The answer is 'No'. Because all database search techniques have some limitations in finding peptides. Besides that, the non-identified peptides can come from some undiscovered organisms as well. On the other hand, human effort to go through the map for locating true positives is not feasible, as already explained. So the only option left is, using existing algorithms to generate labeled samples. But none of them are perfect. However, multiple algorithms usually do not report the same false positives. Therefore we can take common set of two algorithms to make a training set [14, 20]. Therefore we believe our training data generation technique is wisely designed.

Supplementary Note D

In this note we discuss about the experimental stages mentioned in Table 10 of the manuscript. The matching with MS/MS identifications mentioned in this table is based on validation sample 9. We divide the experiments into following stages:

- Stage 1: This is the initial model designed with FC-RNN network with three convolution layers, one fully connected layer, without any pooling layer and state size 4. It gives about 87.55% matching.
- Stage 2: This is the model after retrained on Adjacent feature cases as mentioned in Supplementary note B. It gives about 92.82% matching.
- Stage 3: The initial model was upgraded with max-pooling layer and one more fully connected layer as shown in Figure 7 of the manuscript. The state size was also raised to 8. This raised the matching to about 94.66%.
- Stage 4: Instead of using FC-RNN, we changed the gating mechanism as attention-gated RNN as explained in the Method section. That is, we use Equation (2), instead of Equation (1) while implementing the RNN cells. This process gives about 0.4% improvement.
- Stage 5: So far we have been using peptide features from 8 LC-MS maps coming from two samples. Now we add one more sample which gives 4 additional LC-MS maps for training. Thus the amount of positive sequence in training set is increased by about 60,000. When Stage 4 was trained with this bigger dataset, we get about 95.13% matching.
- Stage 6: Here we just ensemble multiple trained IsoGrouping modules to get the final result. We ensemble four models presented in Table S 1. This gives about 95.46% matching.

Models	Learning Rate	State Size	Last fully connected layer size	Individual Matching
1	0.07	8	128	95.43
2	0.08	10	128	95.22
3	0.08	10	80	95.36
4	0.09	8	128	95.19

Supplementary Table S 1. Models used for Ensemble

We also show a matching vs epoch plot in Figure S 3. We run validation step every 10 minibatch after epoch 95 and select the state with maximum matching.

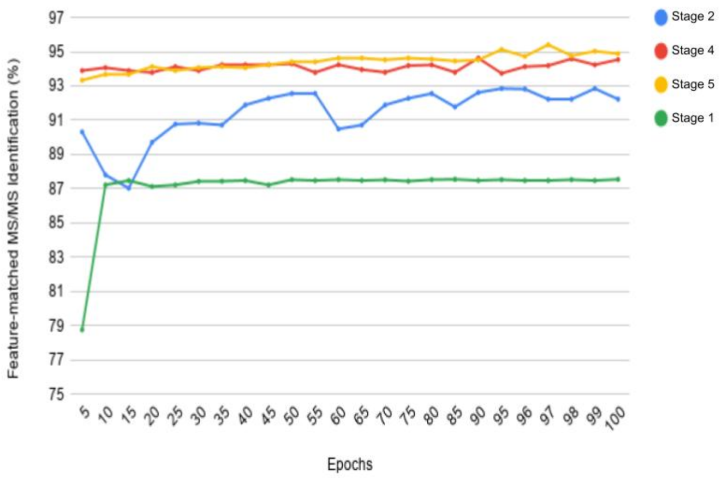

Supplementary Figure S 3. Matching vs epochs plot for validation LC-MS map 9_01

Supplementary Method A

Scanning Algorithm for 'IsoGrouping' Module

We elaborate the scanning procedure of 'IsoGrouping' module step by step using Supplementary Figure S4. Let us consider cluster 'P' in the figure. Each isotope is marked with it's index, starting from 0. The frames are placed at five successive isotopes of the cluster (marked with red colored arrow). The output is generated at last time step which can be one of the frame indexes: 0 to 4. In first round, for instance, it outputs 3. Therefore, we get a feature starting at X m/z, with RT peak t_1, and 4 isotopes. Next, we place the scanning window at the 4th isotope of the cluster, i.e., just the next one. This round sees through 4th to 8th isotopes of the cluster. In this round the output is 4. Therefore, we should extend the counting of isotopes further to see if there are more isotopes belonging to the current feature. So we start another round, but this time it starts from 8th frame (instead of 9th frame). After seeing through 5 next frames, it outputs 1. That means, we find the second feature starting at the 4th isotope and ending at the 9th isotope of the cluster. This scanning continues until all the isotopes of the cluster are seen. Also note that, we apply batch processing to speed up the scanning procedure. Therefore the cluster 'P' and 'Q' are passed together in the same batch to the 'IsoGrouping' module. After each round completes, we run an iteration to see which cluster has become empty, thus discarded from the batch in the next round.

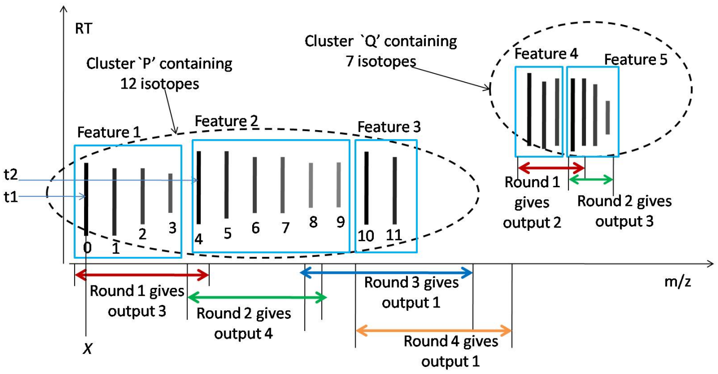

Supplementary Figure S 4. Scanning procedure of 'IsoGrouping' module

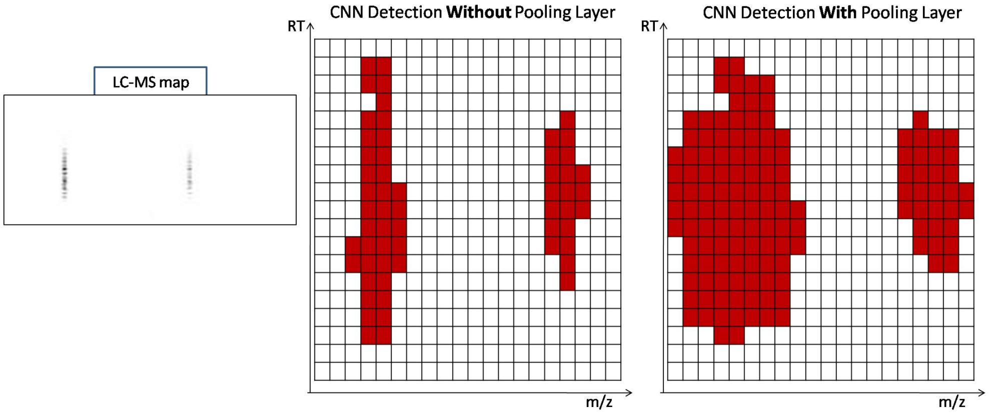

Supplementary Figure S 5. We see the effect of pooling layers in IsoDetecting module. A peptide feature with charge 1 is shown in LC-MS map. When we use pooling layer to detect features, the isotope detections are wider, as shown in the right most image. But if we avoid using pooling layer, then the detections are thin and nicer, as presented in middle image.

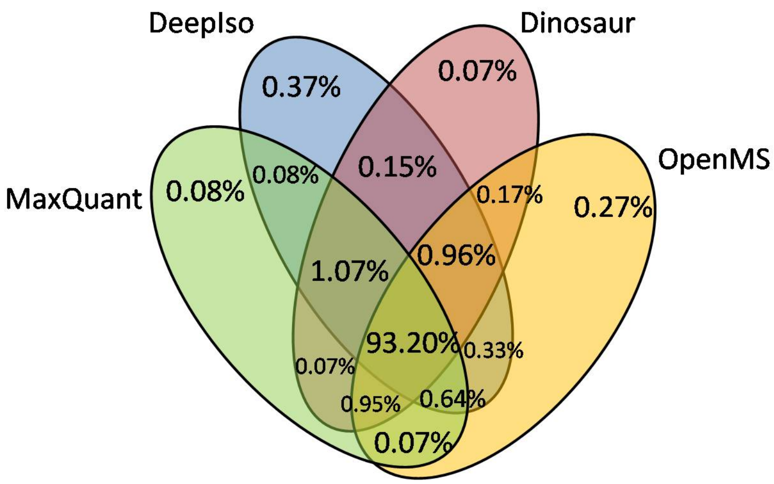

Supplementary Figure S 6. Venn Diagram of feature-matched MS/MS identification by different tools. The blue area shows that DeepIso is capable of finding some peptide features not detected by other tools.

Class (z)	0	1	2	3	4	5
Old architecture (only CNN with fixed interval along RT axis)	85.04%	82.08%	90.96%	86.53%	83.87%	62.18%
Upgraded architecture (both CNN and RNN, with MS-Scans given intervals along RT axis)	96.43%	93.80%	96.98%	98.74%	97.94%	85.86%

Supplementary Table S 2. Better learning (higher class sensitivity) by IsoDetecting module with upgraded architecture.

Supplementary Table S3: The percentage of high confidence MS/MS identified peptides matched with peptide features for different samples

Samples	OpenMS	MaxQuant	Dinosaur	DeepIso
130124_dilA_1_01	94.4704	94.7819	95.8723	96.1838
130124_dilA_1_02	96.1095	95.389	95.6772	96.902
130124_dilA_1_03	96.0727	95.8545	95.9273	96.6545
130124_dilA_1_04	96.1401	95.9971	96.2831	96.8549
Average of sample # 1	95.698175	95.505625	95.939975	96.6488
130124_dilA_2_01	96.2468	95.8651	96.6285	96.6921
130124_dilA_2_02	96.0656	96.2623	96.7869	97.0492
130124_dilA_2_03	96.3715	95.572	96.679	96.4945
130124_dilA_2_04	96.7378	96.1731	96.2359	96.5496
130124_dilA_2_05	96.8474	96.4691	96.7844	96.8474
130124_dilA_2_06	96.867	96.4194	96.9949	97.3146
130124_dilA_2_07	97.0849	96.4512	96.4512	96.7681
Average of sample # 2	96.603	96.17317143	96.65154286	96.8165
130124_dilA_3_01	96.4286	95.7627	96.9128	97.0944
130124_dilA_3_02	96.6875	96.875	97.25	97
130124_dilA_3_03	96.802	96.7405	97.786	97.6015
130124_dilA_3_04	96.8358	96.1791	96.8955	97.0149
130124_dilA_3_05	96.8658	96.9249	97.2206	96.7475
130124_dilA_3_06	97.3072	96.634	97.3072	97.0012
130124_dilA_3_07	96.7895	96.4328	96.195	96.5517
Average of sample # 3	96.81662857	96.507	97.08101429	97.0016
130124_dilA_4_01	96.6187	96.556	96.8691	96.9944
130124_dilA_4_02	96.224	96.0286	96.0286	97.2005
130124_dilA_4_03	96.4901	96.873	97.4474	97.575
130124_dilA_4_04	96.5711	96.2594	97.5062	96.9451
130124_dilA_4_05	97.0428	96.379	96.8014	97.3446
130124_dilA_4_06	97.558	96.4591	97.4969	96.8254
130124_dilA_4_07	97.7724	96.9296	97.652	97.05
Average of sample # 4	96.89672857	96.49781429	97.11451429	97.13357143
130124_dilA_5_01	95.7616	95.9603	96.8874	97.1523
130124_dilA_5_02	95.8413	96.865	96.865	96.801
130124_dilA_5_03	97.1411	96.6439	97.1411	97.4518
130124_dilA_5_04	96.6422	96.0317	97.1306	97.0085
Average of sample # 5	96.34655	96.375225	97.006025	97.1034
130124_dilA_6_01	96.7347	96.5986	96.8707	96.8707
130124_dilA_6_02	96.3542	96.0286	96.4844	97.3958
130124_dilA_6_03	97.0121	96.4399	96.4399	96.9485
130124_dilA_6_04	96.5304	96.4684	96.7162	97.026
Average of sample # 6	96.65785	96.383875	96.6278	97.06025
130124_dilA_7_01	95.3235	95.3235	95.7719	96.1563
130124_dilA_7_02	99.3902	98.7805	99.3902	99.0854
130124_dilA_7_03	99.4083	98.2249	98.2249	99.1124

130124_dilA_7_04	98.892	98.338	98.338	98.338
Average of sample # 7	98.2535	97.666725	97.93125	98.173025
130124_dilA_8_01	99.0066	98.3444	99.0066	98.6755
130124_dilA_8_02	99.6865	99.373	99.6865	99.373
130124_dilA_8_03	98.3607	98.3607	98.6885	99.3443
130124_dilA_8_04	99.0625	99.0625	99.0625	99.375
Average of sample # 8	99.029075	98.78515	99.111025	99.19195
130124_dilA_9_01	96.0682	95.7405	96.4613	96.5269
130124_dilA_9_02	96.8202	96.0415	97.0798	96.8202
130124_dilA_9_03	95.9371	95.9371	96.5269	96.4613
130124_dilA_9_04	96.7516	96.6242	97.1975	97.2611
Average of sample # 9	96.394275	96.085825	96.816375	96.767375
130124_dilA_10_01	98.8095	98.5119	99.4048	99.7024
130124_dilA_10_02	97.2441	98.4252	99.6063	99.2126
130124_dilA_10_03	99.0385	98.3974	98.7179	98.7179
130124_dilA_10_04	99.359	98.7179	99.359	99.359
Average of sample # 10	98.612775	98.5131	99.272	99.247975
130124_dilA_11_01	97.8947	97.5439	96.8421	98.9474
130124_dilA_11_02	98.1618	97.7941	97.7941	98.8971
130124_dilA_11_03	96.7262	97.0238	97.619	97.619
130124_dilA_11_04	97.4684	97.1519	96.519	96.2025
Average of sample # 11	97.562775	97.378425	97.19355	97.9165
130124_dilA_12_01	95.4717	95.4717	95.0943	95.0943
130124_dilA_12_02	97.5806	96.371	97.5806	95.9677
130124_dilA_12_03	97.2468	96.5585	97.1485	96.3618
130124_dilA_12_04	97.0646	95.9883	96.5753	96.9667
Average of sample # 12	96.840925	96.097375	96.599675	96.097625
